# An SINS/GNSS Ground Vehicle Gravimetry Test Based on SGA-WZ02

**DOI:** 10.3390/s150923477

**Published:** 2015-09-16

**Authors:** Ruihang Yu, Shaokun Cai, Meiping Wu, Juliang Cao, Kaidong Zhang

**Affiliations:** College of Mechatronics and Automation, National University of Defense Technology, Changsha 410073, China; E-Mails: yuruihang@nudt.edu.cn (R.Y.); csk527@163.com (S.C.); cjl.nudt@gmail.com (J.C.); kdzhang@263.net (K.Z.)

**Keywords:** SGA-WZ02, SINS/GNSS, ground vehicle gravimetry, FIR filter, internal and external accuracy

## Abstract

In March 2015, a ground vehicle gravimetry test was implemented in eastern Changsha to assess the repeatability and accuracy of ground vehicle SINS/GNSS gravimeter—SGA-WZ02. The gravity system developed by NUDT consisted of a Strapdown Inertial Navigation System (SINS), a Global Navigation Satellite System (GNSS) remote station on test vehicle, a GNSS static master station on the ground, and a data logging subsystem. A south-north profile of 35 km along the highway in eastern Changsha was chosen and four repeated available measure lines were obtained. The average speed of a vehicle is 40 km/h. To assess the external ground gravity disturbances, precise ground gravity data was built by CG-5 precise gravimeter as the reference. Under relative smooth conditions, internal accuracy among repeated lines shows an average agreement at the level of 1.86 mGal for half wavelengths about 1.1 km, and 1.22 mGal for 1.7 km. The root-mean-square (RMS) of difference between calculated gravity data and reference data is about 2.27 mGal/1.1 km, and 1.74 mGal/1.7 km. Not all of the noises caused by vehicle itself and experiments environments were eliminated in the primary results. By means of selecting reasonable filters and improving the GNSS observation conditions, further developments in ground vehicle gravimetry are promising.

## 1. Introduction

Major advances in kinematic gravimetry have been extensively studied during the last few years and successfully applied in many geodesy and geophysical applications [1,2,3,4]. Several kinematic gravimeters with different principles have been developed, such as two-axis stable platform system (e.g., LCR A/M Gravimeter [5,6]), the gimbaled inertial navigation system (e.g., GT-1A, AIRGrav [7,8]), and the SINS (e.g., SISG [9,10,11,12], SGA-WZ01 [13,14,15]). Results of airborne gravity surveys all over the world showed an accuracy of 1–3 mGal with a spatial resolution less than 5 km is available to be obtained [2,8,11].

Considering that the power of the gravity field (especially the short wavelength part) intensifies steadily with a decrease in the altitude, the signal-to-noise ratio (SNR) should increase in the terrestrial system [2]. In recent years, the ground vehicle gravimetry has attracted researchers’ attentions and had a rapid development. In June 2005, a successful ground vehicle gravimetric survey was implemented by Ohio State University in west Montana. When all systems were working at peak performance, better than 1 mGal repeatability in the down component of the gravity disturbance could be obtained, and the standard deviation respecting to the control data was 2–3 mGal [16]. Compared to satellite and airborne gravimetry, the lower velocity of ground vehicle gravimetry should improve the resolution and accuracy of the gravity information. Besides, satellite and airborne gravimetry test need to be carried out very cautiously and thriftily because of financial restrictions. Generally speaking, gravity data is calculated after airborne gravimetry tests are carried out. If by any chance the gravimeter does not work normally and researchers cannot find the mistakes during the test, it is a waste of time and money for this airborne test. Conducting a ground vehicle gravimetry test before the gravimeter applied for airborne gravimetry is helpful to check the gravimeter working status and avoid unnecessary expense. In addition, the disadvantage of airborne gravimetry is that a process of gravity downward continuation is needed to obtain gravity on the surface of the Earth. The process may reduce the resolution and accuracy of observed gravity. Although the traditional static ground gravimetry offers the higher accuracy, it is labor intensive and time consuming to adequately cover the survey region [17]. For certain particular applications in geology and geophysics, it requires the knowledge of a local gravity field with a higher spatial resolution (1–10 km, or even higher), ground vehicle gravimetry will serve as an important role in many particular applications in the near future. 

In principle, the closer the gravimeter is to the earth surface, the stronger the gravity signal is. However, comparing the terrestrial moving vehicle gravimetry with the airborne test, differences between carriers, velocities, altitudes, and trajectories will result in different characteristics of the observables [16]. Although the gravity signal is stronger on the ground than that in airborne gravimetry, the slower moving vehicle causes more complicated disturbances than those in the airborne system. 

The new generation gravimeter SGA-WZ02 was designed by National University of Defense Technology (NUDT) in 2014 [18]. Based on the former generation gravimeter SGA-WZ, SGA-WZ02 has got a further development on external appearance and internal performances. The purpose of the test was assessing the feasibility of this gravimetric system for land vehicle gravity determination and preparing for the forthcoming airborne gravimetry test. In March 2015, a ground vehicle gravimetry test was carried out in eastern Changsha, Hunan province in China. The principle of strapdown terrestrial moving base gravimetry will be briefly introduced in Section 2. This paper shows the test in detail, including the system description in Section 3 and the test description in Section 4. Data processing will be presented in Section 5. After analyzing the differences between ground vehicle and airborne gravimetry, results of ground vehicle test are presented for discussion in Section 6. It is necessary to adjust the airborne methods for the ground vehicle system in order to obtain valuable terrestrial results. And these terrestrial featured methods are a fair way to advise more meaningful suggestions for kinematic gravimetry.

## 2. Method of Strapdown Ground Moving Base Gravimetry

The principle of kinematic gravimetry is based on Newton’s equation of motion in the gravitational field of the earth [1]. In navigation frame (*n*-frame), the model of moving base gravimetry is expressed by the equation [19]

(1) $$g^{n}={\dot{v}}_{e}^{n}-C_{b}^{n}f^{b}+(2\omega_{ie}^{n}+\omega_{en}^{n})\times v_{e}^{n}$$

where $v_{e}^{n}$ and ${\dot{v}}_{e}^{n}$ are the velocity and acceleration of vehicle with respect to the earth, $f^{b}$ is the specific force sensed by accelerometers of a strapdown Inertial Navigation System (INS) in body frame (*b*-frame), $C_{b}^{n}$ is the transformation matrix which rotates $f^{b}$ from *b*-frame to *n*-frame, $\omega_{ie}^{n}$ is angular velocity of the earth respecting to the n-frame and $\omega_{en}^{n}$ is rotation rate of the *n*-frame due to vehicle rate over the ellipsoid, $g^{n}$ is the gravity vector which expressed in *n*-frame.

Utilizing the relative gravimetry method, the gravity disturbance vector needs to be calculated. Considering introducing γ as the normal gravity vector, Equation (1) can be rewritten as

(2) $$\delta g^{n}={\dot{v}}_{e}^{n}-C_{b}^{n}f^{b}+(2\omega_{ie}^{n}+\omega_{en}^{n})\times v_{e}^{n}-\gamma^{n}$$

then $\delta g^{n}$ is the gravity disturbance vector.

Expanding to three pairwise orthogonal directions like north, east and down components, Equation (2) can be rearranged by

(3) $$\begin{array}{l} \delta g_{N}={\dot{v}}_{N}-f_{N}+2\omega_{ie}\cdot v_{E}\cdot \sin L-\frac{v_{N}\cdot v_{D}}{R_{M}+h}+\frac{v_{E}^{2}\cdot \tan L}{R_{N}+h}\\ \delta g_{E}={\dot{v}}_{E}-f_{E}-2\omega_{ie}\cdot (v_{N}\cdot \sin L+v_{D}\cdot \cos L)-\frac{v_{E}\cdot v_{N}\cdot \tan L}{R_{N}+h}-\frac{v_{E}\cdot v_{D}}{R_{M}+h}\\ \delta g_{D}={\dot{v}}_{D}-f_{D}+(2\omega_{ie}\cdot \cos\varphi +\frac{v_{E}}{R_{N}+h})\cdot v_{E}+\frac{v_{N}^{2}}{R_{M}+h}-\gamma \end{array}$$

where $v_{N}$, $v_{E}$ and $v_{D}$ are the north, east and down components of the vehicle velocity from GNSS processing, $f_{N}$, $f_{E}$ and $f_{D}$ are the north, east and down components of the specific force, $R_{M}$ and $R_{N}$ are the meridian and prime vertical radius of curvature, φ, L and h are latitude, longitude, and height in geodetic coordinates. Equation (2) can be computed for both vector and scalar gravimetry. In the last few years, vector and scalar gravimetry has been deeply studied and have achieved plenty of promising results [4,20,21,22,23]. When only considering the vertical component of gravity vector, scalar gravimetry equation can be estimated by the third component of Equation (3). In this paper, only the scalar gravimetry will be discussed in detail.

The basic principle of strapdown ground moving base gravimetry is similar to that of airborne gravimetry. The gravity disturbance vector $\delta g^{n}$ in Equation (2) and the gravity disturbance $\delta g_{D}$ in Equation (3) can be computed by the measurements from three accelerometers of an IMU like $f^{b}$ and the observed quantities from GNSS positioning information such as $\varphi ,h,R_{M},R_{N},\gamma ,v_{e}^{n},{\dot{v}}_{e}^{n},\omega_{ie}^{n},\omega_{en}^{n}$, *etc*. This approach to land vehicle gravimetry is well known as Strapdown Inertial Scalar Gravimetry (SISG) [9,10].

## 3. System Description

SGA-WZ02 system is the new generation strapdown gravimeter designed by NUDT in 2014. This system is composed of three subsystems: a strapdown inertial navigation system, two GNSS receivers (one on the land vehicle, the other on the known-position ground station), and a data-logging subsystem. In this section, SGA-WZ02 will be introduced in three parts: the inertial sensors unit, the electric control unit, and the power supply unit.

**Figure 1:**
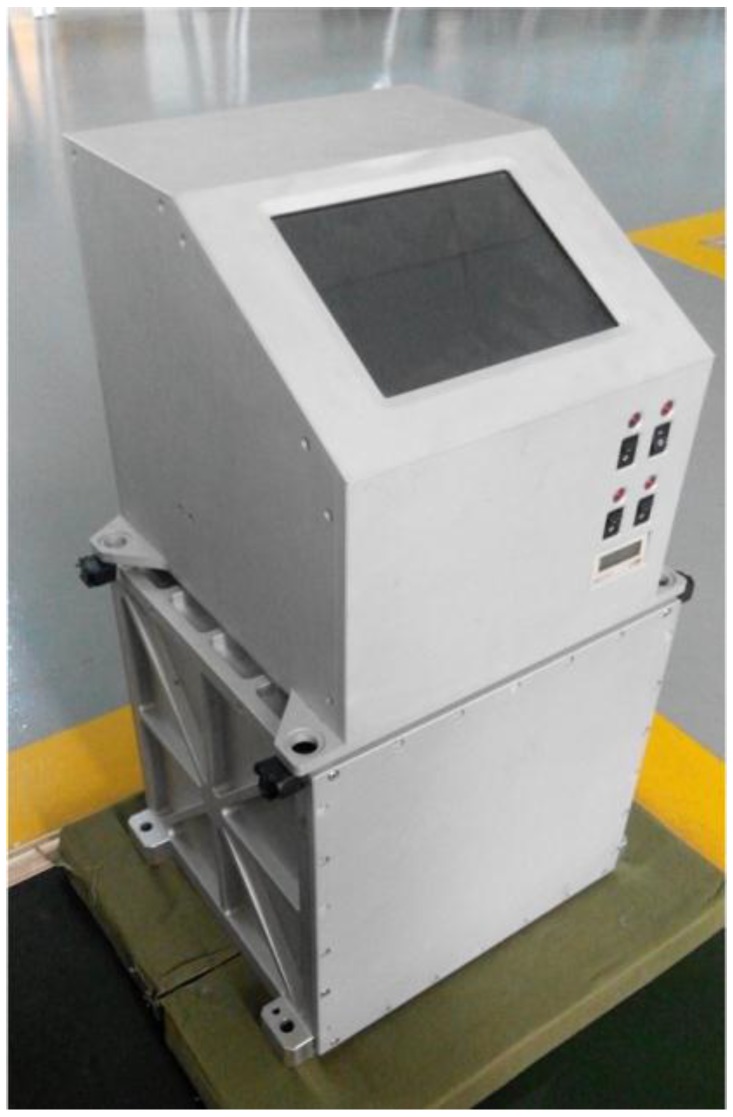
SGA-WZ02 gravimeter.

As shown in Figure 1, the lower part of SGA-WZ02 is the Inertial Measurement Unit (IMU) box and the electric control unit located on the IMU box. This IMU consists of one triad of quartz flexibility accelerometers and three pairwise orthogonal navigation-grade Ring Laser Gyroscopes (RLG). The stability of the three navigation-grade RLGs equipped on SGA-WZ02 was ±0.004°/h and the random noise was ±0.002°/$\sqrt{\text{h}}$. Errors caused by ambient temperature changing were always the important factors which evidently restricted the increase of the accelerometer’s accuracy. A temperature control system with the precision at a level of 0.02 °C was designed for the specific force system, considering it was sensitive to change of the working environment temperature. With this precise thermal control system applied in SGA-WZ02, a stability of accelerometers came to a level of 0.6 mGal/day.

The electric controlling unit consists of a liquid crystal display, four temperature controllers, a GNSS receiver, and a data-logging subsystem. One can make a primary judgment from the information displayed on screen whether the system is working normally or not. NovAtel GNSS receivers were applied to both GNSS master station and remote station. The data-logging subsystem was used to record all inertial data from data-collecting circuit boards at a rate of 200 Hz and all GNSS data from GNSS receivers at a rate of 2 Hz. Avoiding the logging system working at overheating status, an electric mini-fan was installed to let the heat out of the unit.

An Uninterrupted Power Supply (UPS) was used for the whole system, which ensured the system working continuously in case of external power sources switching or other unexpected circumstances like sudden power-down accidents. Usually the system was supplied by automotive electrical source, and the UPS was the backup choice. Besides, another advantage of this UPS is compactness and portability, which makes it convenient for transport.

## 4. Test Description

**Figure 2:**
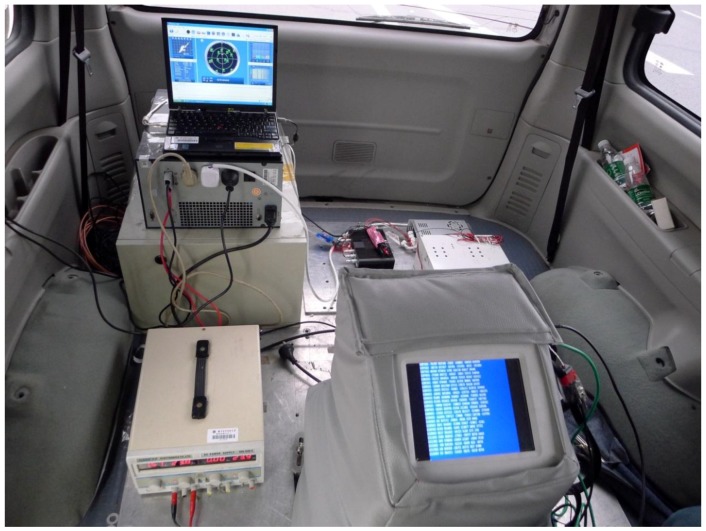
Working state of SGA-WZ02 on the test vehicle.

The land vehicle gravimetry test was carried out on a highway in eastern Changsha, Hunan province in March, 2015. Distance of the tested highway in south-north direction was about 40 km. Both the well-maintained highway and light traffic volume were helpful to implement the test smoothly and effectively. The interior look of the system is shown in Figure 2.

**Figure 3:**
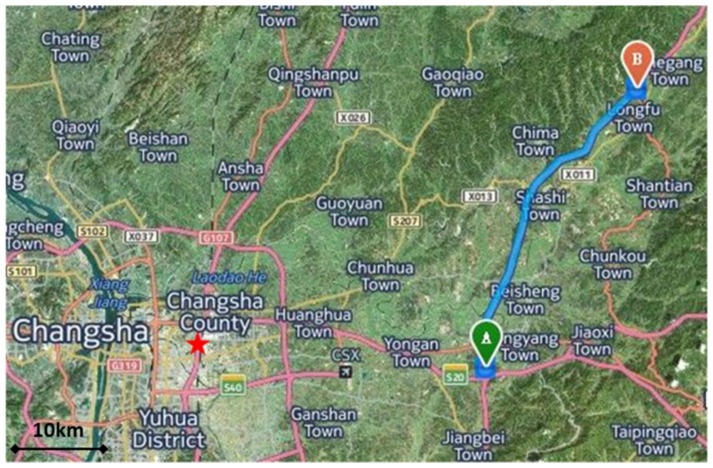
Map of test trajectories in eastern Changsha. The red star was the position of GNSS master station. Blue line from A to B was the test trajectory.

The survey map of trajectories is shown in Figure 3. The GNSS master station equipped with a NovAtel receiver, was located on the roof of a nine floors laboratory building (red star in Figure 3) where it could provide ideal static observation conditions. The maximum baseline distance was 52 km, while the minimum baseline distance was 36 km. The GNSS remote station was equipped on the vehicle, with a magnet fixing the dual-frequency antenna on the vehicle roof.

The test consisted of four repeated measurement lines along the south-north direction highway. The available length of each profile was about 35 km and the vehicle was driving at an average speed of 40 km/h. Four repeat profiles were measured in this test. Unfortunately, the second line lost the GNSS signal for about 10 min because the vehicle refueled at the gas station during the test. In the following results analysis section, this period during the GNSS signal lost has been removed and more attention will be focused on the available GNSS signal periods.

## 5. Data Processing

### 5.1. External Gravity Reference Processing

To obtain a meaningful assessment of the gravimetry capability of the SGA-WZ02 system as accurate as possible, precise along-track gravity disturbances are necessary to be built as the reference data [16]. A high accuracy relative gravity test was carried out along the tested highway using CG-5 gravimeter produced by the Scintrex^TM^ Company (Concord, ON, Canada), which was able to sense the difference of gravity signals less than 0.01 mGal. The CG-5 ground gravimeter is easy to operate and is able to obtain a reliable result in 6 min at each point. Since the predicted purpose of land vehicle gravimetry was providing the performances at the level of 1–2 mGal for spatial resolutions about 2 km, the sensing accuracy of CG-5 gravimeter was accurate enough as the external gravity disturbances reference. On March 31, about 34 positions along the tested highway of approximately 1 km intervals were measured using this gravimeter. Both the CG-5 gravimeter and the IMU have the identical height. So it would not cause the gravity differences because there was no height difference between CG-5 and the IMU box of SGA-WZ02. While the CG-5 gravimeter was measuring the gravity, the GNSS was also collecting the data at the same time. In the following the procedure of reference gravity data processing will be presented step by step.

Step 1: Recording the measure results sensed by CG-5 gravimeter *R_0_* at the known absolute gravity base position *G_0_*, connecting the relationships between *R_0_* and *G_0_*;
Step 2: Recording the positioning results *L_i_*, *λ_i_*, *h_i_* from the GNSS receiver and the measure results sensed by CG-5 gravimeter *R_i_* (*i* = 1, 2, 3,…, 34) along the tested highway, the average interval between every two adjacent points was about 1 km;
Step 3: Calculating absolute gravity *G_i_* of each point obtained by Step 2 using the relationships between *R_0_* and *G_0_*, *i.e.*, $G_{i}=G_{0}+R_{0}-R_{i},(i=1,2,3,...,34)$;
Step 4: Interpolating the calculated *G_i_* using splining method to obtain high resolution absolute gravity reference $G^{\prime }$;
Step 5: Calculating normal gravity γ which was the function of latitude L and height *h* with the same resolution of $G^{\prime }$ along the tested highway;
Step 6: Calculating the gravity disturbances $\Delta g=G^{\prime }-\gamma$.

Thus, the high accuracy gravity data has been obtained as the reference for the following external accuracy assessment.

### 5.2. GNSS Data Processing

Taking advantage of the SISG method, errors in the GNSS-derived vertical accelerations have a direct effect on the accuracy of the gravity disturbance estimates [10]. Typically, double differenced carrier phase measurements method was used for GNSS processing in this test.

As presented in the introduction, the slower moving vehicle causes more complicated errors than those in the airborne system. Among these disturbances, errors caused by non-ideal observation conditions play a major part and need to be analyzed and eliminated carefully. 

Characters of ground test and airborne test are different in many aspects. In this section, GNSS data comparison between airborne tests and this ground vehicle test was coupled, presented, and analyzed. Making a comparison in this way, a typical ground vehicle processing method is expected to help improve the accuracy of GNSS results.

In airborne gravimetry, an airplane flies at a high altitude where the observation conditions were ideal for GNSS working. With the help of automatic pilot, the plane can maintain at a relatively stable height and keep a uniform velocity cruising status.

**Figure 4:**
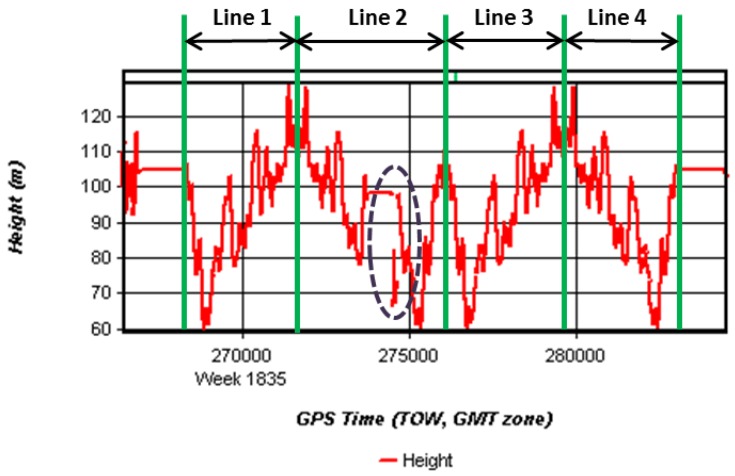
Height profile plot of ground gravimetry. The topographic profile of the measuring line was relatively rough with the peak-to-peak value of about 50 m. Line 1~4 were four repeated lines in this test. There was a data gap around 274,470 and 274,650 s of the week.

**Figure 5:**
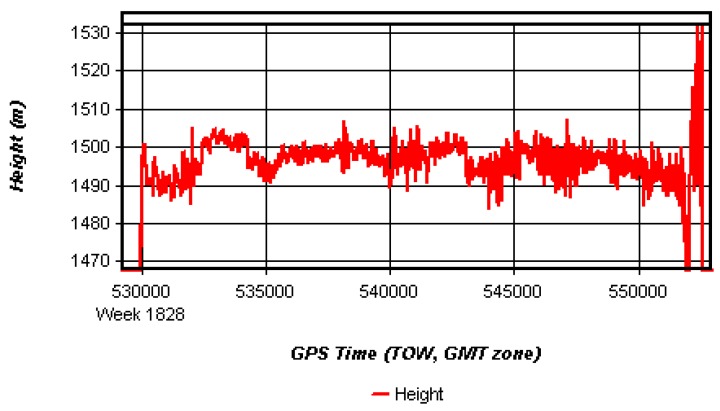
Height profile plot of airborne gravimetry. In this figure, the flight altitude was about 1500 m above the sea-level, meanwhile, 400 m above the ground. The variation around setting altitude was about ±8 m.

The height profiles of measuring lines are shown in Figure 4 and Figure 5. These two figures show that unlike the airborne test, the ground survey routes may have ups and downs or turns frequently while the plane can fly straight and steadily using automatic pilot system. The GNSS logging system may have data gaps due to signal blocked by trees, trucks, and or some other unexpected situations. In Figure 4, it is noted that there was a data gap around 274,470 and 274,650 s of the week. It was because the test vehicle refueled at the gas station for a while, which severely degraded the quality of the observables, even output the wrong results. These GNSS processing data results had to be corrected. First, check whether there were obvious errors or not in the primary calculated height data. With a vehicle running along the road, the measured heights varied with the road terrain which changed smoothly. If there were abrupt changes in the heights, all these abnormal results should be checked and found. Second, eliminate the abnormal results and fill the data gaps using interpolation methods. For example, during the test vehicle refueling at the gas station, the vehicle stayed stopped while the heights jumped from 98 m to 80 m. Considering the parked status, it is reasonable to eliminate these obvious wrong heights results (about 70–80 m). Then the authors found the time before vehicle refueled t_1_ = 274,460 s and the time after vehicle refueled t_2_ = 274,660 s. After obtaining the positions of t_1_ and t_2_, linear interpolation method was selected to fill up the position gaps.

**Figure 6:**
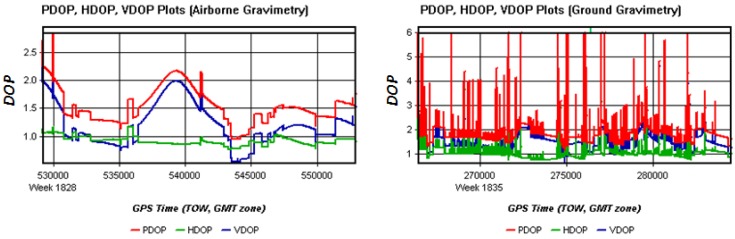
DOP plots of airborne and ground tests. In airborne gravimetry, three DOPs change smoothly in the range of 0.5 to 2.5, however in the ground test, the DOPs change frequently and sensitively in the range of 0.5 to 6.

**Figure 7:**
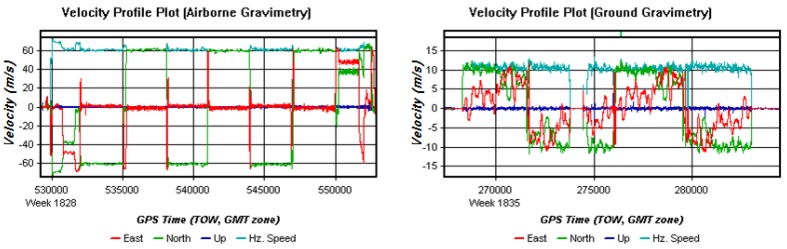
Velocity profile plots of airborne and ground test.

The Dilution of Precision (DOP) Plots of airborne and ground gravimetry are shown in Figure 6. The larger DOP is, the poorer GNSS quality is. The larger and more mutable DOPs in the ground test also imply that a ground vehicle test has worse conditions than an airborne test. In the normal case, with so many cars and traffic lights on the road and frequently happened with turns and brakes during the ground moving test, it is difficult to keep a steady velocity. These complicated conditions will bring severe challenges to ground vehicle gravimetry. Fortunately, the traffic pressure was not so high on the day when the ground test was conducted. The average speed was about 11 m/s with a little variation. Velocity profiles of airborne and ground gravimetry are shown in Figure 7.

**Figure 8:**
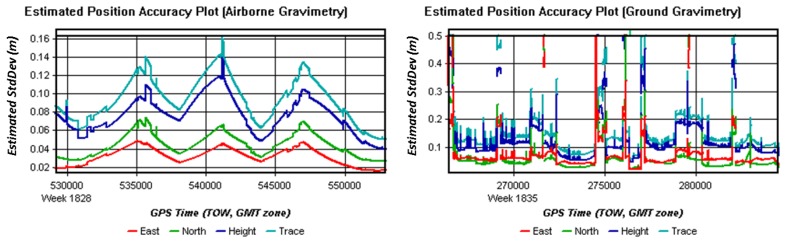
Estimated position accuracy plots of airborne and ground test.

**Figure 9:**
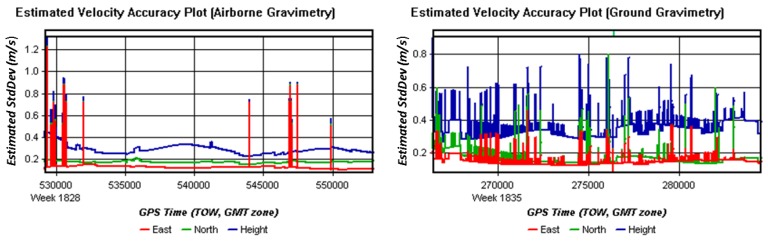
Estimated velocity accuracy plots of airborne and ground test.

As the estimated position and velocity accuracy plots show in Figure 8 and Figure 9, position errors in the ground vehicle test has larger and more frequent changes than those in the airborne test, and velocity errors showed the same features to position errors.

A detailed comparison table of differences between GNSS data in ground test and in airborne gravimetry test was presented in Table 1.

**Table 1:** GNSS results comparison of airborne test and ground test.

Items	Airborne Gravimetry	Ground Gravimetry
**Altitude**	1500 m	60~130 m
**Velocity**	60 m/s	11 m/s
**Estimated Position Accuracy**	$\delta p_{N}$: 0.03~0.07 m	$\delta p_{N}$: 0.04~0.08 m
	$\delta p_{E}$: 0.02~0.05 m	$\delta p_{E}$: 0.06~0.10 m
	$\delta p_{D}$: 0.05~0.10 m	$\delta p_{D}$: 0.08~0.14 m
	δp: 0.06~0.14 m	δp: 0.12~0.18 m
**Estimated Velocity Accuracy**	$\delta v_{N}$: 0.15~0.20 m/s	$\delta v_{N}$: 0.15~0.25 m/s
	$\delta v_{E}$: 0.10~0.15 m/s	$\delta v_{E}$: 0.15~0.20 m/s
	$\delta v_{D}$: 0.22~0.36 m/s	$\delta v_{D}$: 0.28~0.40 m/s
**DOP**	0.8~2.3	0.8~5.8
**Number of Satellites**	7~8	4~7
**Forward/Reverse or Combined Separation**	−0.08~0.14 m	−0.50~0.60 m
**Forward/Reverse or Combined RMS**	0~0.10 m	0~0.60 m

Summarizing the analysis of the differences above, we can draw a primary conclusion that the observation conditions in ground vehicle gravimetry are worse than those in airborne gravimetry. It shows mainly in two aspects: one is the larger errors in positioning results; the other is more mutable signal quality during the tests. In addition, comparing the average baseline distances between airborne and ground vehicle gravimetry, it appears that the long baseline is not the major factor which degrades the accuracy evidently, one of the most important factors is the complicated observation conditions such as blockings of GNSS signal in ground test. The purpose of GNSS data processing was acquiring precise positions, velocities, and accelerations which were quite crucial to calculate the precise gravity disturbances. It is necessary to explore ways and means to overcome the above difficulties. To reduce errors as much as possible, several effective measures were taken as follows:

(1) Wrong Data Checking: Check the primary data, and correct the obvious wrong results. In ground vehicle gravimetry, the GNSS signal was often blocked by trucks or trees nearby. It was insufficient for obtaining correct positioning results under these situations. To make matters worse, it may even get obvious incorrect results when less than four satellites can be observed. For example, as mentioned in Figure 4, during the period of 274,470 and 274,650 s, the vehicle was stopped and refueled at a gas station. The GNSS antenna covered by the roof of gas station resulted in the height jumped from 98 m to 80 m.

(2) Data Gaps Interpolating: Check whether the results are consistent. If not, interpolate the data gaps using nearby data with reasonable interpolation methods. According to different driving statuses, choose the linear or splining interpolation method to get the consistent positioning results.

(3) Forward and Reverse Directions Computation: GNSS data can be calculated after the test finished. In this test, double differenced carrier phase method was selected to process the GNSS data. Calculating the original results in both forward direction and reverse direction using the time-related GNSS data, there were differences between these two directions. Because different directions would mainly result in different ionosphere effects and different carrier phase ambiguity solutions, and all these differences would output different positioning results. Combining two directions results with reasonable weights that minimize the errors, optimal positioning results can be acquired.

The data quality had a perceptible improvement after the GNSS modified data adopting the measures above. It appears that velocity of about 0.28~0.40 m/s and positions of better than 0.18 m can be achieved in this ground gravimetry test. By means of GNSS data processing procedure, the positioning results can be applied on integrated navigation computation to determine the gravity disturbances.

### 5.3. Gravity Disturbance Determining

**Figure 10:**
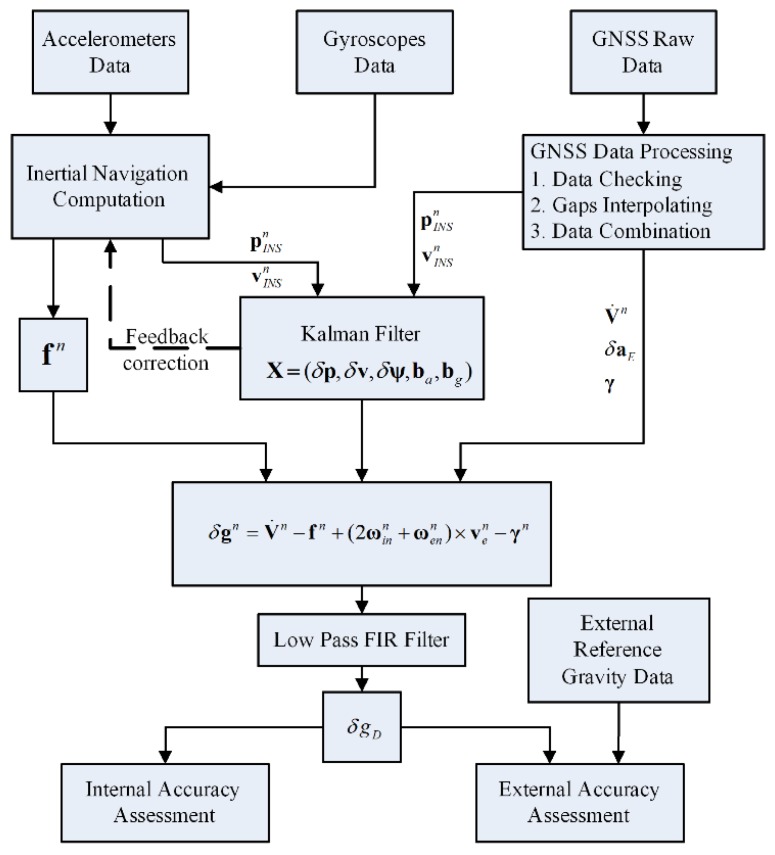
Data processing flow chart.

As the whole data processing flow chart shows in Figure 10, implementing the SINS/GNSS integrated navigation computation with a 15-state Kalman Filter, the specific force $f^{n}$ can be calculated from $f^{b}$ by transformation matrix $C_{b}^{n}$. The position and velocity of vehicle can be obtained from the former part of GNSS data processing, and acceleration ${\dot{v}}^{n}$, Eötvös correction $\delta a_{E}$, normal gravity γ can also be calculated in this processing part. Then the gravity disturbance can be acquired by Equation (3). Determining the gravity disturbance should be processed carefully because the noise is much larger and more complicated than the real gravity signal. Considering the low SNR character of gravity disturbances, a low-pass Finite Impulse Response (FIR) filter was used to remove high frequency noise and extract the useful gravity information from polluted signals [24]. For example, if a 200 s FIR low-pass filter was used, the average speed of a land vehicle is 11 m/s, then a spatial resolution about 1.1 km can be achieved.

## 6. Results and Discussion

Generally speaking, there are two methods to evaluate the results: internal accuracy and external accuracy. The internal accuracy evaluating repeated measure lines represents the repetitiveness of the gravimeter which sensed several times on the same test trajectory [25]. On the other hand, the external accuracy evaluates the difference between gravimeter calculated data and external reference data. It represents the objectiveness and reliability of the gravimeter compared with the true gravity data [26]. Fortunately, we have both repeated measure lines for internal accuracy assessment and precise gravity reference data for external accuracy evaluation.

The internal accuracy of repeat measure line *j* can be calculated by Equations (4)–(6) [24].

(4) $$\varepsilon_{j}=\pm \sqrt{\frac{\sum_{i=1}^{n}\delta_{ij}^{2}}{n}},(j=1,2,...,m)$$

(5) $$\delta_{ij}=\delta g_{ij}-\delta g_{i},(i=1,2,...,n;j=1,2,...,m)$$

(6) $$\delta g_{i}=\frac{\sum_{j=1}^{m}\delta g_{ij}}{m},(i=1,2,...,n)$$

where $\delta_{ij}$ is the difference between $\delta g_{ij}$ and $\delta g_{i}$, $\delta g_{ij}$ is the observed value of point *i* along repeat measure line *j*, $\delta g_{i}$ is the average value of point *i* of all repeat measure lines, *n* is the number of observed points in each repeat measure line, and *m* is the number of the repeat measure lines.

The total internal accuracy of all repeat measure lines can be calculated by Equation (7).

(7) $$\varepsilon =\pm \sqrt{\frac{\sum_{j=1}^{m}\sum_{i=1}^{n}\delta_{ij}^{2}}{m\times n}}$$

The external accuracy (using reference gravity data) can be calculated by Equation (8).

(8) $$\sigma =\pm \sqrt{\frac{\sum_{k=1}^{n}\omega_{k}^{2}}{n}}$$

(9) $$\omega_{k}=\delta g_{k}-\delta g_{ck},(k=1,2,...,n)$$

where $\omega_{k}$ is the difference between $\delta g_{k}$ and $\delta g_{ck}$, $\delta g_{k}$ is the observed value of point k, $\delta g_{ck}$ is the control value of point k, and *n* is the number of observed points.

**Figure 11:**
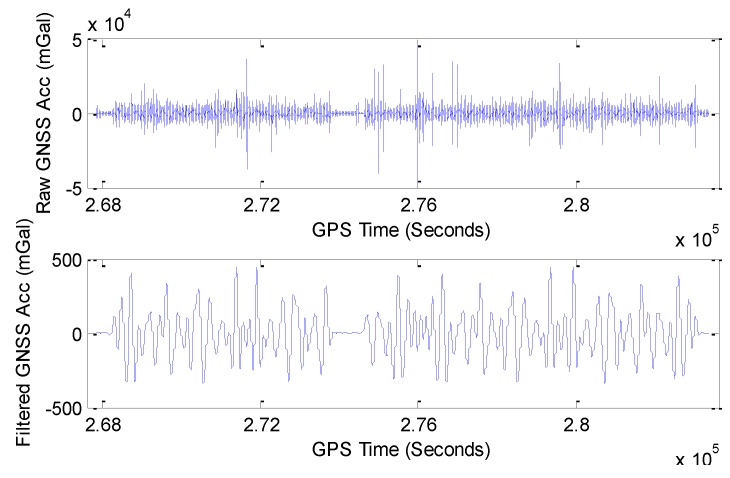
Plots of raw and 200 s filtered GNSS accelerations.

A useful gravity signal is hiding deeply in the raw observation accelerations. To get further low-frequency accelerations for gravity determination, an FIR filter with hundreds of seconds should be applied. During the whole test, plots of raw GNSS acceleration and 200 s filtered GNSS acceleration are shown in Figure 11.

**Figure 12:**
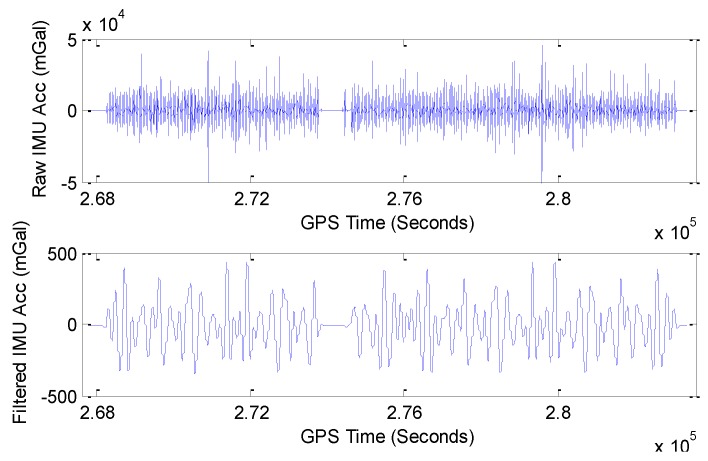
Plots of raw and 200 s filtered IMU accelerations.

Figure 12 shows the plots of raw IMU acceleration and 200 s filtered IMU acceleration.

In this paper, four normal-tested measure lines were used to evaluate the internal accuracy and external accuracy. There is a middle point between gravity accuracy and spatial resolution. The shorter length of the FIR filter used, the higher resolution and less accuracy acquired. The resolution and filter length should be selected carefully to get optimal results.

**Figure 13:**
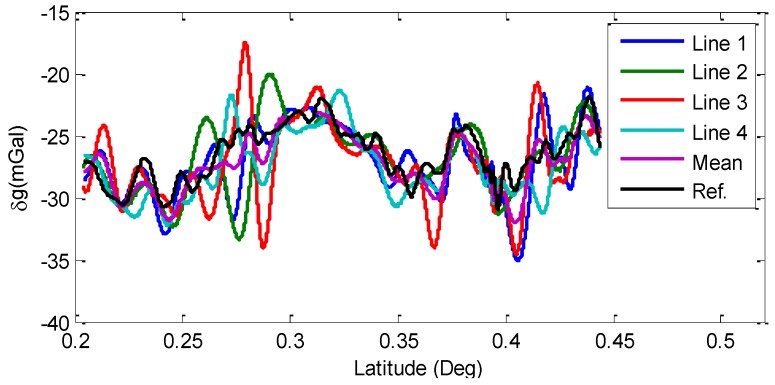
Filtered gravity disturbances with 200 s FIR filter.

During the refueling at the gas station in Line 2, GNSS signal was blocked for several minutes. After removing the refueling period, results with 200 s FIR filter are shown in Figure 13. In this paper, the X-axis “Latitude” was expressed based on relative coordinates.

The statistics of the results with 200 s FIR filter corresponding to Figure 13 are shown in Table 2. From Table 2, we can see that the total internal RMS of repeat measure lines is 1.86 mGal after the 200 s low-pass filter calculating, and the total external RMS of measure lines is 2.27 mGal.

**Table 2:** Accuracies of 200 s FIR filtered gravity disturbances (Units: mGal).

Items		Max	Min	Mean	RMS (Each Line)	Total RMS
Internal					$\varepsilon_{j}$	ε
	Line 1	4.24	−4.09	0.02	1.44	**1.86**
	Line 2	6.64	−6.68	0.33	2.03	
	Line 3	8.02	−6.82	−0.08	2.17	
	Line 4	5.85	−5.80	−0.26	1.70	
External					$\sigma_{j}$	σ
	Line 1	4.55	−6.61	−0.63	2.15	**2.27**
	Line 2	4.85	−8.93	−0.32	2.27	
	Line 3	7.30	−8.97	−0.72	2.71	
	Line 4	4.29	−5.55	−0.91	1.88	

**Figure 14:**
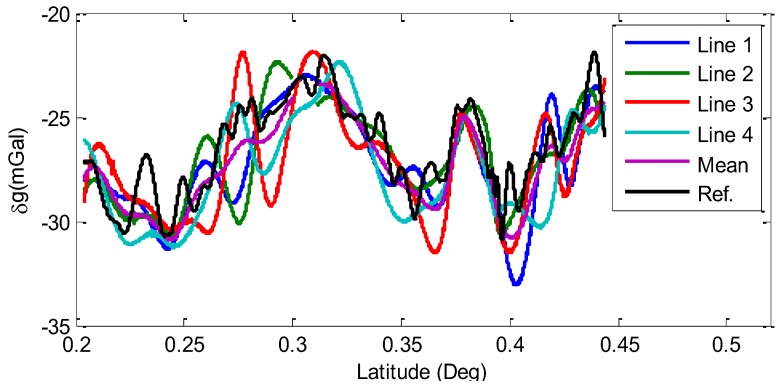
Filtered gravity disturbances with 300 s FIR filter.

Figure 14 shows the results with 300 s FIR filter and gravity disturbances with 300 s FIR filtered results are shown in Table 3.

**Table 3:** Accuracies of 300 s FIR filtered gravity disturbances (Units: mGal).

Items		Max	Min	Mean	RMS (Each Line)	Total RMS
Internal Accuracy					$\varepsilon_{j}$	ε
	Line 1	2.49	−2.50	−0.02	0.98	**1.22**
	Line 2	3.30	−3.79	0.39	1.25	
	Line 3	4.35	−3.39	−0.10	1.40	
	Line 4	4.35	−3.39	−0.27	1.21	
External Accuracy					$\sigma_{j}$	σ
	Line 1	2.48	−5.39	−0.78	1.77	**1.74**
	Line2	2.53	−5.69	−0.37	1.57	
	Line 3	2.88	−4.52	−0.86	1.89	
	Line 4	2.53	−4.23	−1.03	1.73	

Corresponding to the vehicle velocity of 11 m/s, 200 s filter length matches the spatial resolution of 1.1 km and 300 s for 1.6 km. From Table 3, the total internal RMS of repeat lines is 1.22 mGal and the external RMS of measure lines is the maximum of 1.89 mGal and the average of 1.74 mGal after the 300 s low-pass filter calculating.

Table 2 and Table 3 show the statistics of the results by calculating four repeated measure lines. During each test period of four trajectories, there may be different situations happening making the test conditions different from each other. Pairwise internal accuracy comparing trajectories is helpful to preliminary assessment of the test conditions.

Table 4 shows the pairwise comparing between four measure lines. Lines 1 and 3 are both driving from south to north while Lines 2 and 4 are in the opposite direction. In this table, we can see that internal accuracy has a few differences between each pair of two measure lines. According to Table 4, it seems that Lines 1 and 2 have more similar conditions than the others because this pair has the minimum RMS. RMS between Lines 2 and 3 seemed to be the largest gap in conditions between these two lines. It seemed that the test conditions had slightly changed during the whole test period.

**Table 4:** Pairwise internal accuracy comparing between four measure lines (Units: mGal).

Filter Lengths	Items	Max	Min	Mean	RMS (*ε*)
**200S**	Line 1–Line 2	3.09	−3.21	−0.15	**1.19**
	Line 1–Line 3	4.60	−4.61	0.05	**1.41**
	Line 1–Line 4	4.80	−4.95	0.11	**1.39**
	Line 2–Line 3	6.66	−7.33	0.20	**1.99**
	Line 2–Line 4	4.17	−5.17	0.30	**1.49**
	Line 3–Line 4	4.90	−2.55	0.13	**1.43**
**300S**	Line 1–Line 2	1.44	−1.98	−0.21	**0.68**
	Line 1–Line 3	2.45	−3.29	0.02	**0.97**
	Line 1–Line 4	2.81	−2.40	0.12	**0.96**
	Line 2–Line 3	3.33	−4.06	0.25	**1.24**
	Line 2–Line 4	2.34	−2.85	0.33	**1.02**
	Line 3–Line 4	2.65	−1.85	0.13	**0.96**

**Figure 15:**
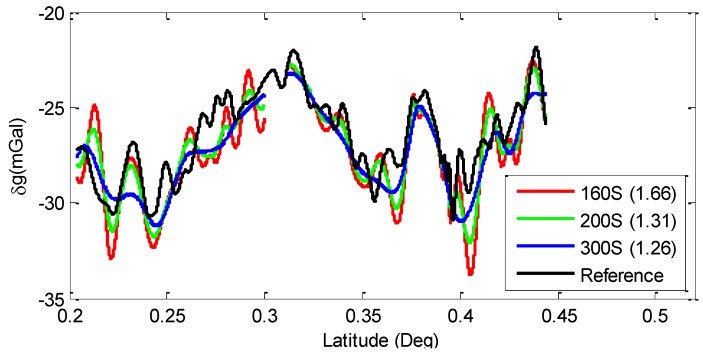
Reference compared with average disturbances using different filter lengths.

Generally speaking, every time we tested the trajectory, there were always special circumstances happening that made the test conditions not all the same all the way. Compared to each line assessment, the average disturbances of repeated lines will present more accurate details. Different lengths of FIR filters (such as 160 s, 200 s and 300 s) were used to calculate the disturbances. Difference between average disturbances and reference data is shown in Figure 15.

From Figure 15, we can see that with 300 s filter computed, the RMS of external accuracy is 1.26 mGal, while 200 s turning to 1.31 mGal and 160 s filter reaching to 1.66 mGal. Although the 300 s filtered line (blue) gets the best accuracy, it seems too long of a filter process period loses many particulars. Meanwhile, the 160 s filtered line (red) has too many sharp wave crests and hollows. Contrasting these three lines to the reference data, the 200 s filtered result (green line) seems to be inosculating a more similar trend and expressing more similar details than the others.

All the figures above have shown the primary results under different filtering conditions. The overall trend of gravity disturbance calculated agrees well with reference gravity data. These figures verified the objectiveness of the calculated data and the reliability of SGA-WZ02 gravimeter. Results of internal accuracy and external accuracy implied that SGA-WZ02 was capable of obtaining the ground data about 1.86 mGal internal accuracy and 2.27 mGal external accuracy at a level of 1.1 km resolution.

However, some errors still exist in the results. The calculated disturbance varies around the real reference data even has a sharp oscillation. The reason may be the vehicle braking suddenly or turning during this period. The measurement lines consist of two south-north direction lines and two opposite direction lines which separate with each other by about 20 m. The frequently varied traffic conditions increased the difficulty of driving smoothly at a uniform velocity. Besides, the hilly area along the tested highway decreased the GNSS data quality which would also degrade the gravity accuracy evidently. Furthermore, characteristics of errors caused by vehicle and internal IMU parts are not studied completely and deeply. Errors still exist deeply behind the true gravity information. Further research on vehicle gravimetry error analysis and elimination is expected to start as soon as possible. Besides, since vector gravimetry has already been studied for years, the ground vector gravimetry test should be implemented and analyzed in the future.

## 7. Conclusions

A ground vehicle gravimetry test was carried out in eastern Changsha using the new generation Strapdown gravimeter SGA-WZ02 designed by NUDT in 2015. The purpose of the test was to assess the feasibility of this gravimetric system for land vehicle gravity determination and to prepare for the forthcoming airborne gravimetry test. Primary results show that SGA-WZ02 is capable of achieving the ground gravity about 1.86 mGal internal accuracy at a level of 1.1km resolution and 1.22 mGal at 1.7 km resolution. The external accuracy comparing gravimeter calculated data with external reference data is about 2 mGal. Results above have verified the objectiveness of the calculated data and the reliability of SGA-WZ02 gravimeter. Based on the former generation SGA-WZ, performance of SGA-WZ02 has provided a further development. This system can be used for ground moving base gravimetry or other applications in geodesy and geophysical survey activities. In a normal case, with so many cars and traffic lights on the road, it is difficult to keep a steady velocity with frequent turns and brakes. These complicated conditions will bring severe challenges to ground vehicle gravimetry. Considering the complicated conditions in ground vehicle gravimetry, especially under sub-optimal GNSS observation conditions, more attention will be paid to the accuracy improvements of vehicle acceleration extracting, error analysis, and vector gravimetry in the research going forward.

## References

[1] Schwarz K.P., Wei M., Sünkel H., Marson I. (1995). Some unsolved problems in airborne gravimetry. Gravity and Geoid.

[2] Kwon J.H. (2000). Airborne Vector Gravimetry Using GPS/INS. Ph.D. Thesis.

[3] Zhang K. (2007). Research on the Methods of Airborne Gravimetry Based on SINS/DGPS. Ph.D. Thesis.

[4] Senobari M.S. (2010). New results in airborne vector gravimetry using strapdown INS/DGPS. J. Geod..

[5] LaCoste L., Ford J., Bowles R., Archer K. (1982). Gravity measurements in an airplane using state-of-the-art navigation and altimetry. Geophysics.

[6] Forsberg R., Olesen A., Keller K., Møller M., Gidskehaug A., Solheim D. Airborne gravity and geoid surveys in the arctic and baltic seas. Proceedings of the International Symposium on Kinematic Systems in Geodesy, Geomatics and Navigation (KIS-2001).

[7] Ferguson S.T., Hammada Y., Sideris M. (2002). Experiences with AIRGrav: Results from a new airborne gravimeter. Gravity, Geoid and Geodynamics 2000.

[8] Studinger M., Bell R., Frearson N. (2008). Comparison of AIRGrav and GT-1A airborne gravimeters for research applications. Geophysics.

[9] Wei M., Schwarz K.P. (1998). Flight test results from a strapdown airborne gravity system. J. Geod..

[10] Glennie C., Schwarz K.P. (1999). A comparison and analysis of airborne gravimetry results from two strapdown inertial/DGPS systems. J. Geod..

[11] Bruton A.M. (2000). Improving the Accuracy and Resolution of SINS/DGPS Airborne Gravimetry. Ph.D. Thesis.

[12] Glennie C.L., Schwarz K.P., Bruton A.M., Forsberg R., Olesen A.V., Keller K. (2000). A comparison of stable platform and strapdown airborne gravity. J. Geod..

[13] Cai S., Zhang K., Wu M., Huang Y. (2012). Long-term stability of the SGA-WZ strapdown airborne gravimeter. Sensors.

[14] Huang Y., Olesen A.V., Wu M., Zhang K. (2012). SGA-WZ: A new strapdown airborne gravimeter. Sensors.

[15] Zhao L., Forsberg R., Wu M., Olesen A., Zhang K., Cao J. (2015). A flight test of the strapdown airborne gravimeter SGA-WZ in greenland. Sensors.

[16] Li X., Jekeli C. Ground-vehicle INS/GPS vector gravimetry assessment using repeated traverses in montana. Proceedings of the 1st international symposium of the international gravity field service.

[17] Li X. (2007). Moving Base INS/GPS Vector Gravimetry on a Land Vehicle. Ph.D. Thesis.

[18] Cai S., Wu M., Zhang K., Cao J. (2015). Application of EMD in data processing of dynamic gravimatry. Hydrogr. Surv. Charting.

[19] Titterton D., Weston J.L. (2004). Strapdown Inertial Navigation Technology.

[20] Jekeli C. (1994). Airborne vector gravimetry using precise, position-aided inertial measurement units. Bull. Géod..

[21] Kwon J.H., Jekeli C. (2001). A new approach for airborne vector gravimetry using GPS/INS. J. Geod..

[22] Forsberg R., Olesen A.V., Keller K., Sideris M. (2002). Airborne gravity survey of the north Greenland continental shelf. Gravity, Geoid and Geodynamics 2000.

[23] Li X. (2011). Strapdown INS/DGPS airborne gravimetry tests in the gulf of mexico. J. Geod..

[24] Cai S., Zhang K., Wu M. (2013). Improving airborne strapdown vector gravimetry using stabilized horizontal components. J. Appl. Geophys..

[25] Cai S., Wu M., Zhang K., Cao J., Tuo Z., Huang Y. (2013). The first airborne scalar gravimetry system based on SINS/DGPS in China. Sci. Chin. Earth Sci..

[26] Guo Z.-H., Xiong S.-Q., Zhou J.-X., Zhou X.-H. (2008). The research on quality evaluation method of test repeat lines in airborne gravity survey. Chin. J. Geophys..

